# A Rare Case of Staphylococcal Toxic Shock Syndrome in a Neonate

**DOI:** 10.1155/2022/8111620

**Published:** 2022-05-31

**Authors:** Nipun Shrestha, Alisha Joshi, Yumiko Hayashi, Dhruba Shrestha, Bhim Gopal Dhoubhadel

**Affiliations:** ^1^Department of Paediatrics, Siddhi Memorial Hospital, Bhaktapur, Nepal; ^2^Department of Clinical Medicine, Institute of Tropical Medicine, Nagasaki University, Nagasaki, Japan; ^3^Department of Respiratory Infections, Institute of Tropical Medicine, Nagasaki University, Nagasaki, Japan; ^4^School of Tropical Medicine and Global Health, Nagasaki University, Nagasaki, Japan

## Abstract

*Staphylococcus* toxic shock syndrome (TSS) is not well described in neonates. The present criteria for diagnosis of TSS have not yet been validated in neonates. Here, we present a case of a 13-day-old female baby who presented with acute kidney injury (AKI). She had a pus-draining lesion on the head, and the pus grew *Staphylococcus aureus*. Based on the clinical criteria of fever, desquamation, hypotension, and AKI and laboratory criteria of absence of growth of any organisms in blood and cerebrospinal fluid, we diagnosed the case as TSS. She was treated with antibiotics, oxygen, and fluids, along with inotropic support and mechanical ventilation, and she recovered fully and was discharged on day 17 of admission. As there is no single test to diagnose TSS and it is uncommon in neonates, physicians should be familiar with the clinical presentation of the disease to make early diagnosis.

## 1. Introduction

Toxic shock syndrome (TSS) is an acute, toxin-mediated, and life-threatening illness. It is characterized by high fever, erythematous rash, hypotension, multiorgan involvement (involving at least three or more organ systems), and desquamation of skin 1-2 weeks after the onset of the acute illness [1]. Other associated symptoms may include myalgia, diarrhea, vomiting, headache, and nonfocal neurological abnormalities [2]. Although most cases of TSS are associated with menstrual causes, 6–36% cases are associated with nonmenstrual causes [3, 4]. Nonmenstrual TSS is associated with *Staphylococcus aureus* infections at various sites, such as cutaneous and subcutaneous lesions, surgical wound infections, postpartum infections, and deep abscesses [5].

Although children and adults with any focal complications of staphylococcal infection or colonization are at risk of developing TSS, very few cases are reported in children [4, 6]. TSS is diagnosed based on a set of clinical criteria established by the Centers for Disease Control and Prevention (CDC) (Table 1) [7]; however, this set of diagnostic criteria has never been validated in neonates [6]. In the neonatal period, when the disease severity of such illnesses is mild and the babies do not meet the criteria of TSS, the syndrome of systemic exanthem, thrombocytopenia, and fever is called neonatal toxic shock syndrome (TSS)-like exanthematous disease (NTED) [8, 9].

Here, we present a neonate with features suggestive of TSS and discuss the challenges of diagnosis, treatment, and management of complications in a resource-limited setting.

## 2. Case Presentation

A 13-day-old female neonate was admitted with fever for 2 days and poor feeding with decreased urine output for 1 day. She had not passed urine for 8 hours. On examination, the baby was lethargic, the axillary temperature was 102°F, breathing was shallow with a respiration rate of 28/min, the heart rate was 164/min with a feeble pulse, the capillary refill time (CRT) was prolonged (>3 sec), oxygen saturation (SpO_2_) was 99% in atmospheric air, and blood pressure was 72/37 mmHg.

She was admitted to the neonatal intensive care unit (NICU). After taking blood for investigations, intravenous fluid and empirical antibiotics (inj cefotaxime 50 mg/kg/dose and inj gentamicin 5 mg/kg/day) were administered. The blood reports showed signs of infection with increased leucocyte count (18600/mm^3^) and increased C-reactive protein (CRP) (48 mg/dL). Arterial blood gas analysis (ABG) showed respiratory acidosis. Random blood sugar level was high (375 mg/dL) which did not decrease even after decrease in glucose infusion rate; therefore, inj regular insulin was started at a dose of 0.05 unit/kg/hour as a continuous infusion.

The baby did not pass urine even after adequate fluid resuscitation. The initial renal function test (RFT) report showed acute kidney injury (AKI) (urea 390 mg/dL, creatinine 3.6 mg/dL, sodium 182 mmol/L, and potassium 8.1 mmol/L), and hence, inj gentamycin was stopped. Serum urea and creatinine level increased rapidly within 2 days of admission. The urea level was 330 mg/dL, and the creatinine level was 11.3 mg/dL. The baby developed generalised body swelling. A pediatric nephrologist was consulted for the rapidly evolving AKI. After evaluating the status of the baby, peritoneal dialysis was postponed until next evaluation. After 6 hours, she did not show any signs of improvement. Blood pressure was on the lower side with a prolonged CRT (>3 seconds). Inj dopamine was started at a dose of 10 microgram/kg/min, and with renal drug dose adjustment, inj meropenem at a dose of 20 mg/kg/day was added. The child also experienced one episode of focal seizure, which was not controlled with inj midazolam (0.1 mg/kg/dose); therefore, inj phenytoin was administered with a loading dose of 20 mg/kg, followed by 5 mg/kg/day. On day 3 of admission, the child started to develop sclerema and inj ofloxacin (10 mg/kg/day) was added.

On day 4 of admission, the child experienced sudden apnea with oxygen desaturation and bradycardia for which she had to be intubated and was kept under a mechanical ventilator. COVID-19 infection was ruled out. Lumbar puncture was performed; the CSF examination showed a total count of 7 cells with 100% lymphocytes, a glucose level of 47 mg/dL, and a protein level of 119 mg/dL. There was no bacterial growth in blood and cerebrospinal fluid cultures. She also experienced focal seizure on the same day for which inj levetiracetam was added at a dose of 20 mg/kg/day. Platelet-rich plasma (PRP) transfusion was performed for the low platelet count (35000/mm^3^). Total fluid intake was managed according to the renal regimen. She gradually showed signs of improvement; hence, inj dopamine and inj levetiracetam were gradually tapered and stopped on day 5 of admission. Extubation was performed on day 7 of admission; thereafter, fever also subsided.

**Figure 1:**
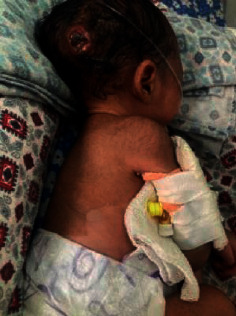
A pus-draining lesion on the scalp and skin desquamation on the trunk.

**Figure 2:**
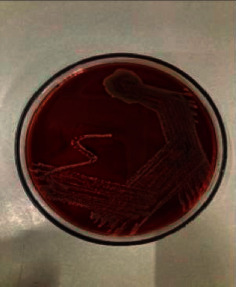
Whitish colonies of *Staphylococcus aureus* in a 5% sheep blood agar plate.

**Figure 3:**
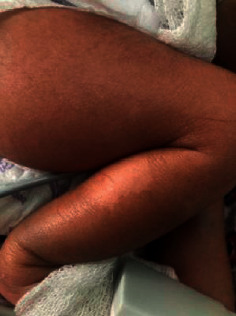
Skin desquamation on the right leg.

On day 9 of admission, a nodular lesion was noted on the occipital region of the scalp from which pus was draining (Figure 1). The pus culture showed *Staphylococcus aureus* (Figure 2). The isolate was sensitive to methicillin and commonly used antibiotics, such as cefotaxime, cefpodoxime, clindamycin, cloxacillin, erythromycin, gentamycin, ofloxacin, piperacillin/tazobactam, vancomycin, amoxycillin/ clavulanic acid, and ceftazidime, and it was resistant to cotrimoxazole. Skin desquamation was noted over the trunk and the legs on day 10 of admission (Figures 1 and 3); however, no mucosal involvement was seen. A diagnosis of staphylococcal toxic shock syndrome was made based on fever, thrombocytopenia, AKI, hypotension, skin desquamation, and the pus culture isolate. Inj meropenem and inj ofloxacin were administered for 14 days. She made a full recovery and was discharged on day 17 of admission.

## 3. Discussion

TSS is rarely diagnosed in neonates. This 13-day-old baby presented with fever, hypotension, oliguria, poor sucking, and somnolence. Lab reports revealed thrombocytopenia and elevated serum creatinine levels; later, during the hospital course, she developed the desquamation of skin on the whole body. No diffuse macular erythematous rash was noted, but a nodular lesion with pus with culture identification of *Staphylococcus aureus* was found on the occipital region. When we tried to fit in the CDC criteria of TSS, this case met 4 of 5 clinical criteria and laboratory criteria of culture results. Therefore, according to the criteria, the case is a probable case of TSS. However, we should keep in mind that these criteria were set by the CDC for epidemiological surveillance, and they have not yet been validated in neonates [6].

Staphylococcal TSS should be suspected in any healthy individuals with the rapid onset of fever, rash, hypotension, and multiorgan system involvement [7]. As no single diagnostic test is available, we have to rely on clinical and laboratory criteria. The diagnosis of TSS is generally made on the basis of the CDC criteria [7]. However, these criteria should not be used to exclude a case that is highly suspicious of toxic shock syndrome, even if all criteria are not met [7]. TSS is a particularly difficult syndrome to diagnose because it mimics other diseases and may have nonspecific initial presentations [10]. The initial features may resemble septic shock. The signs of infection may be moderate to severe, and involvement of multiple organs can be life-threatening. Table 2 shows some of the salient differences between septic shock and toxic shock syndrome.

TSS in neonates is relatively a newly recognized neonatal infectious disease, although it was first described by Todd et al. in 1978 in previously healthy children [13, 14]. There are limited data describing epidemiology, management, and outcomes for children with TSS [1, 15]. A study performed by Aliza et al. reported most patients had fever, vomiting, and rash; however, 48% of patients had no rash [16]. A study over a 17-year period reported 50 cases of TSS in children ≤5 years of age with more than half of cases in children ≤2 years of age, and most of these were associated with nonsurgical cutaneous lesions. The overall case-fatality ratio was 4% [4]. There are reports of neonates developing fever, exanthema, and thrombocytopenia in the NICU in Japan since 1990s; these NTED cases were milder in severity than TSS and did not fulfil the criteria for the diagnosis of TSS [8, 9].

Our baby presented with AKI. AKI in TSS has been reported before; it was due to rhabdomyolysis [17]. There are no separate definitions of AKI in adults and children. Its diagnosis is based on a rise in serum creatinine or a decrease in the urine output. A significant rise in serum creatinine is seen only after 48 to 72 hours of renal insult and is detected when more than 50% of the glomerular filtration rate (GFR) is lost [18]. The new biomarkers such as neutrophil gelatinase lipoprotein (NGAL), cystatin C (CysC), and kidney injury molecule-1 (KIM-1) are beneficial in early detection of AKI in neonates, including premature babies [18, 19]. However, there are limited studies regarding these new biomarkers in neonates and they may not be available in resource-limited regions. Currently, peritoneal dialysis is the modality of choice of treatment for worsening AKI in infants [18]. There is no definitive level of creatinine, at which peritoneal dialysis is recommended. In our case, serum creatinine reached as high as 11.36 mg/dL but resolved without peritoneal dialysis.

TSS occurs because of the production of toxic shock syndrome toxin-1 (TSST-1) by *Staphylococcus aureus* that induces severe immunologically mediated inflammatory responses that affect multiple organ systems and thereby mimics various diseases. TSST-1 and staphylococcal enterotoxins *A*, *B*, and *C* act as superantigens, which directly activate *T* cells and result in massive cytokine release [1]. Children colonized with TSST-1 producing *S*. *aureus* with insufficient antibody titers are at a greater risk of developing TSS [10]. Lack of detectable antibodies to TSS-1 in serum shows susceptibility to TSS [20].

Prompt recognition and timely treatment of TSS are important because the clinical course can deteriorate quickly and the outcome depends on the early aggressive treatment strategies [1, 15, 16]. Treatment of the underlying cause, avoiding exposure to nephrotic medications, appropriate fluid management, timely intubation, and timely use of inotropes may play a very important role in good outcomes of TSS cases. Besides, the use of antibiotics and conservative surgical debridement aimed at eradicating the source of infection and thus the source of toxin production [21].

In conclusion, although we think this case was of TSS, the present CDC diagnostic criteria were not perfectly matched and classified it as a diagnosis of probable TSS. There is a need for the validation of these criteria in neonates. The clinical characteristics of TSS may be vague and nonspecific, especially in neonates. As no single diagnostic test is available to identify TSS at present and it is rarely diagnosed in neonates, physicians should be aware that it can occur in neonates too. Early recognition, effective treatment, and timely management of the complications are essential for good outcomes.

## Figures and Tables

**Table 1:** Clinical criteria for diagnosis of staphylococcal toxic shock syndrome [7].

Clinical criteria
(1) Fever ≥38.9°C (102°F)
(2) Rash: diffuse macular erythroderma
(3) Desquamation: 1 to 2 weeks after onset of rash
(4) Hypotension: for adults, systolic blood pressure ≤90 mmHg; for children <16 years of age, systolic blood pressure less than 5th percentile by age
(5) Multisystem involvement (3 or more of the following organ systems)
Gastrointestinal: vomiting or diarrhea at the onset of illness
Muscular: severe myalgia or creatinine phosphokinase elevation >2 times the upper limit of normal
Mucous membrane: vaginal, oropharyngeal, or conjunctival hyperemia
Renal: blood urea nitrogen or serum creatinine >2 times the upper limit of normal or pyuria (>5 leukocytes/high-power field) in the absence of urinary tract infection
Hepatic: bilirubin or transaminases >2 times the upper limit of normal
Hematologic: platelets <100,000/micro-L
Central nervous system: disorientation or alterations in consciousness without focal neurologic signs when fever and hypotension are absent
Laboratory criteria
Cultures (blood or cerebrospinal fluid) negative for alternative pathogens (blood cultures may be positive for *Staphylococcus aureus*)
Serologic tests negative (if obtained) for Rocky Mountain spotted fever, leptospirosis, or measles
Case classification
Probable case: a case that meets the laboratory criteria and four of the five clinical criteria
Confirmed case: a case that meets the laboratory criteria and all five of the clinical criteria, including desquamation (unless the patient dies before desquamation occurs)

^∗^The above criteria were established for epidemiologic surveillance; they should not be used to exclude a case that is highly suspicious for toxic shock syndrome, even if all criteria are not met.

**Table 2:** Salient differences between septic shock and toxic shock syndrome [1, 5, 7, 11, 12].

Septic shock	Toxic shock syndrome
(i) It may be caused by a dysregulated host response to the infection	(i) It is caused by exotoxin produced by *Staphylococcus aureus*
(ii) Multiorgan failure is possible	(ii) Reversible renal failure is frequently reported
(iii) No skin desquamation occurs	(iii) Skin desquamation is common
(iv) Rash or soft tissue necrosis is not a common finding	(iv) Soft tissue necrosis and skin discoloration may be present
(v) Thrombocytopenia although may be present, it is not included in the SIRS criteria for the diagnosis	(v) Thrombocytopenia is a criterion for the diagnosis

## Data Availability

Data related to this study can be obtained upon reasonable request to the corresponding author.

## Abbreviations

AKI: Acute kidney injury
CDC: Centers for Disease Control and Prevention, USA
CRP: C-reactive protein
CRT: Capillary refill time
Inj: Injection
NTED: Neonatal toxic shock syndrome-like exanthematous disease
TSS: Toxic shock syndrome
TSST-1: Toxic shock syndrome toxin-1.
